# Life in fluctuating environments

**DOI:** 10.1098/rstb.2019.0454

**Published:** 2020-11-02

**Authors:** Joey R. Bernhardt, Mary I. O'Connor, Jennifer M. Sunday, Andrew Gonzalez

**Affiliations:** 1 Department of Aquatic Ecology, Eawag: Swiss Federal Institute of Aquatic Science and Technology, Überlandstrasse 133, 8600 Dübendorf, Switzerland; 2 Department of Zoology and Biodiversity Research Centre, University of British Columbia, 6270 University Boulevard, Vancouver, Canada V6T 1Z4; 3 Department of Biology, Quebec Centre for Biodiversity Science, McGill University, Montreal, Canada H3A 1B1

**Keywords:** feedback, feedforward, environmental noise, environmental variability, phenotypic plasticity, anticipatory systems

## Abstract

Variability in the environment defines the structure and dynamics of all living systems, from organisms to ecosystems. Species have evolved traits and strategies that allow them to detect, exploit and predict the changing environment. These traits allow organisms to maintain steady internal conditions required for physiological functioning through feedback mechanisms that allow internal conditions to remain at or near a set-point despite a fluctuating environment. In addition to feedback, many organisms have evolved feedforward processes, which allow them to adjust in anticipation of an expected future state of the environment. Here we provide a framework describing how feedback and feedforward mechanisms operating within organisms can generate effects across scales of organization, and how they allow living systems to persist in fluctuating environments. Daily, seasonal and multi-year cycles provide cues that organisms use to anticipate changes in physiologically relevant environmental conditions. Using feedforward mechanisms, organisms can exploit correlations in environmental variables to prepare for anticipated future changes. Strategies to obtain, store and act on information about the conditional nature of future events are advantageous and are evidenced in widespread phenotypes such as circadian clocks, social behaviour, diapause and migrations. Humans are altering the ways in which the environment fluctuates, causing correlations between environmental variables to become decoupled, decreasing the reliability of cues. Human-induced environmental change is also altering sensory environments and the ability of organisms to detect cues. Recognizing that living systems combine feedback and feedforward processes is essential to understanding their responses to current and future regimes of environmental fluctuations.

This article is part of the theme issue ‘Integrative research perspectives on marine conservation’.

## 1. Introduction

Global change is characterized by trends, cycles and variability in the environment on land and in the oceans. Rates of change in climate [1], habitat loss and fragmentation [2], chemical contamination [3,4], nutrient deposition and biocide application are high, raising concern among scientists about the capacity of living systems to adapt and persist in the face of these changes [5–7]. While mean conditions are changing, so too are the patterns of variability around the trends in the mean [8,9]. Long-term changes in the variance and autocorrelation of environmental fluctuations can affect biodiversity and ecosystem processes [10–14]. We address here the task of developing an integrated understanding of how individuals, populations and communities respond to, mitigate and adapt to environmental fluctuations.

**Figure 1.**
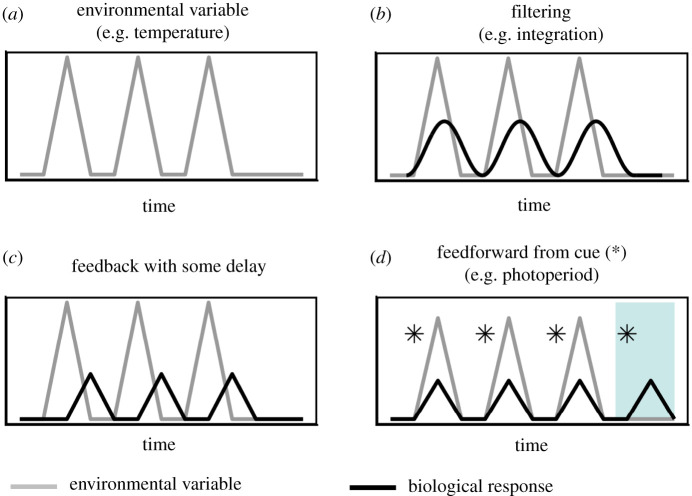
Biological systems filter, integrate, respond to and anticipate environmental variation. (*a*) Environments are characterized by regular fluctuations in environmental variables (e.g. temperature, light, precipitation, oxygen). (*b*) Living systems (individuals, populations, communities) filter or integrate environmental fluctuations (grey line), thereby smoothing environmental time series (black line). As a result, time series of biological or ecological processes that integrate environmental variation tend to have more low-frequency noise compared to the environmental variable itself (i.e. they become ‘redder’ [see box 1]) as they are translated through biological systems. (*c*) Feedback mechanisms (i.e. those that respond to their own internal state) allow organisms to respond to environmental fluctuations, either through dynamical feedback processes or evolutionary adaptations, but only after the fluctuation has occurred. Therefore, there is an inevitable time lag in the response. (*d*) Feedforward mechanisms are signal- or cue-based and use the state of the environment to anticipate environmental change. In nature, such systems may be adaptive because the correlation between the cue and the likely future environmental state allows organisms to employ a response that increases fitness in fluctuating environments. By anticipating the likely change in environmental state, the lag that is inherent in (*b*) and (*c*) is reduced. The disadvantage with feedforward mechanisms is that if the cue (*) becomes uncorrelated with the future environmental state (i.e. the cue becomes an inaccurate indicator of the future state) then organisms may initiate an anticipatory behaviour that is no longer beneficial in the later selective environment (blue shaded area in *d*).

Perhaps the simplest way for variation in the environment to affect living systems (any biological system with some level of autonomy—a cell, an organism, a population, a mutualism, etc.) is for living systems to track their environment as it varies (figure 1*a,b*, box 1). Considering an organism in an environment with fluctuating temperatures as an example, biological rates such as photosynthesis or reproduction may increase or decrease because of the temperature dependence of metabolic rates. Similarly, fluctuations in food or water availability may directly affect demographic rates and therefore population dynamics. Many examples of biological variation have been explained this way—from insect population cycles responding with a time lag under varying weather conditions [19,20] to population cycles in lynx and hares [21] to the abundance of commercially valuable fish [22,23].

**Figure 2.**
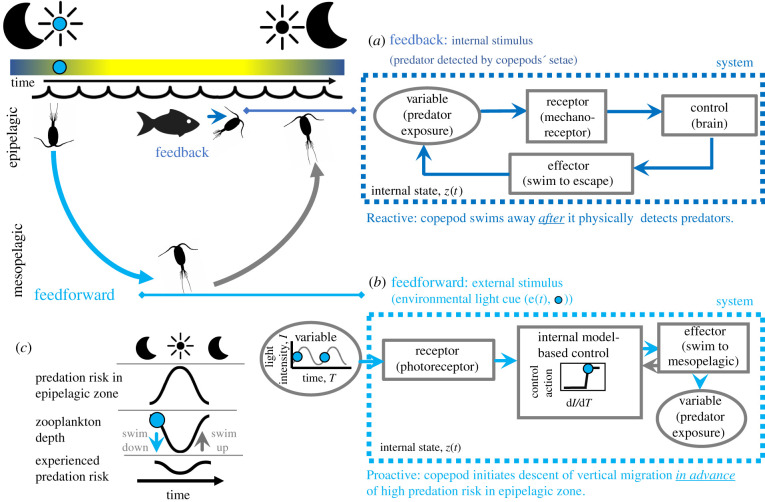
Feedback and feedforward processes allow living systems to persist in fluctuating environments by allowing them to minimize fluctuations in fitness-defining variables (e.g. predation risk). Copepods and other zooplankton combine feedback and feedforward processes to avoid predation in sunlit surface waters. Copepods feed in the surface waters (epipelagic zone) where phytoplankton is abundant. However, feeding in sunlit, illuminated surface waters exposes copepods to visual predators. (*a*) Copepods can detect predators via their setae, which are mechanoreceptors. When setae bend, this may elicit a neurophysiological response in the brain (the controller), triggering the copepod to swim away (effector). This escape behaviour is a type of feedback process—detecting predators causes copepods to move away from predators until they are no longer detectable. Feedback processes are reactive in that they occur after changes in their internal state, *z*(*t*), such as bending of setae owing to predator presence, have occurred. (*b*) Feedforward processes, such as diel vertical migrations, occur when organisms respond to some external environmental cue, *e*(*t*), here indicated by a light blue circle, to control an internal variable such as predator exposure. An internal model allows organisms to ‘pull the future into the present’ [25] by acting, in the present, on some cue that is correlated to a likely future environmental state. In this case, the change in light (d*I*/d*T*), which precedes periods of high predation risk during day time, is used as a predictive cue to adjust depth (i.e. light-cued vertical migration) in order to escape predation. This feedforward mechanism allows zooplankton to move to deeper depths (the mesopelagic zone) proactively at sunrise, before surface waters (epipelagic zone) become sunlit and predation risk by visual predators increases (*c*). Feedforward mechanisms may be combined with feedback mechanisms that allow organisms to respond to predators after they are detected. In (*a,b*), light blue arrows correspond to the feedforward process while dark blue arrows correspond to the feedback process. The grey arrow back from ‘effector’ to ‘internal model’ in (*b*) indicates that internal models can change as the environment changes, a feature of general adaptive systems (GAS). These changes to internal models may occur via learning or other mechanisms by which organisms update their internal models or of those of their offspring.

Another mechanism by which organisms and populations react to a fluctuating environment is through a range of *feedback* mechanisms—when organisms, populations and communities respond to deviations in their internal conditions from a set-point or steady state (box 2, figure 2). Feedbacks are reactive processes, requiring that organisms' or populations’ internal conditions have changed enough to elicit a response in physiological, demographic or other ecological rates (figures 1*c* and 2*a*, box 2, figure 1*a*). As we discuss below, feedback mechanisms can either be adaptive in the evolutionary sense, or can emerge from physical constraints in a system, in both cases increasing persistence of living systems over the long term.

Box 1. **Quantifying the predictability of environmental fluctuations from an organism's eye view.**

Here we consider the predictability of an environment from the perspective of organisms living in fluctuating environments. We consider two types of predictability: (1) predictability that emerges from temporal autocorrelation in a single environmental variable (e.g. how similar today's temperature is to tomorrow's temperature); (2) predictability that emerges from correlations between two or more distinct environmental variables (e.g. temperature and oxygen, or photoperiod and temperature).

**(1) Temporal autocorrelation increases predictability**

Regular variation in a time series lends itself to prediction (box 1, figure 1*a*). The most straightforward case is temporal autocorrelation without a time lag, in which the conditions at any time point are very similar to the conditions in the previous time point. From the perspective of an organism, the greater the temporal autocorrelation, the greater the predictability of the environment because there is an increased probability of having long runs of above- or below-average conditions. Autocorrelation can be visualized using a correlogram, which quantifies the dependence of values in a time series on values preceding them (at a distance of *k* lags) (box 1, figure 1*b*).

Time series can present predictable variation through periodic variation, where conditions at a given time are most similar to conditions at some time in the past—perhaps in the previous year. Environmental variation can incorporate multiple periods of variation (box 1, figure 1*c*), and different biological processes or different organisms may cue on or focus on one or a few aspects of a complex temporal structure. Temporal autocorrelation increases as the dominance of variation at low frequencies increases.

Observing temporal variation and distinguishing patterns that might lend themselves to prediction by biological systems can be challenging and require appropriate statistical analyses. Spectral analysis is a method to decompose variation in time series into component frequencies, allowing one to determine how much of the variance in the time series is associated with different frequencies (box 1, figure 1*b*). The Fourier transform [15] can be used to shift between the time domain (i.e. time on the *x*-axis) and the frequency domain (i.e. frequency on the *x*-axis) (box 1, figure 1*a–c*). In this way, any time series can be rewritten as a sum of sine waves, each with its own amplitude and phase. The spectrum, a plot of variance versus frequency, provides a standardized map of the relative contributions of the underlying components of a time series (e.g. yearly versus daily cycles, box 1, figure 1*c*). When there are smaller amplitudes and less variance at high frequencies (short periods) compared with low frequencies (long periods), the environment can be considered as being more predictable based on the current state, because there is an increased probability of having long sequences of above or below the average conditions. In this way, the predictability of the environment can be understood as the slope of the relationship between variance and frequency. Specifically, if variance scales with frequency (*f*) according to an inverse power law, 1/*f*, then the predictability of the time series can be quantified by the value of the slope, *β*. Where *β* = 0, this indicates that the time series is composed of an equal mix of cyclic components at all frequencies, and the variance (or power) is constant with respect to frequency (also called *white noise*) and random through time. As the value of *β* increases it reveals autocorrelation at longer time scales, which means greater predictability because the time series is dominated by variation at lower frequencies. By analogy with light, we say that temporal variation is reddened when it is dominated by low-frequency (long period) cycles, and 0.5 < *β* < 1.5.

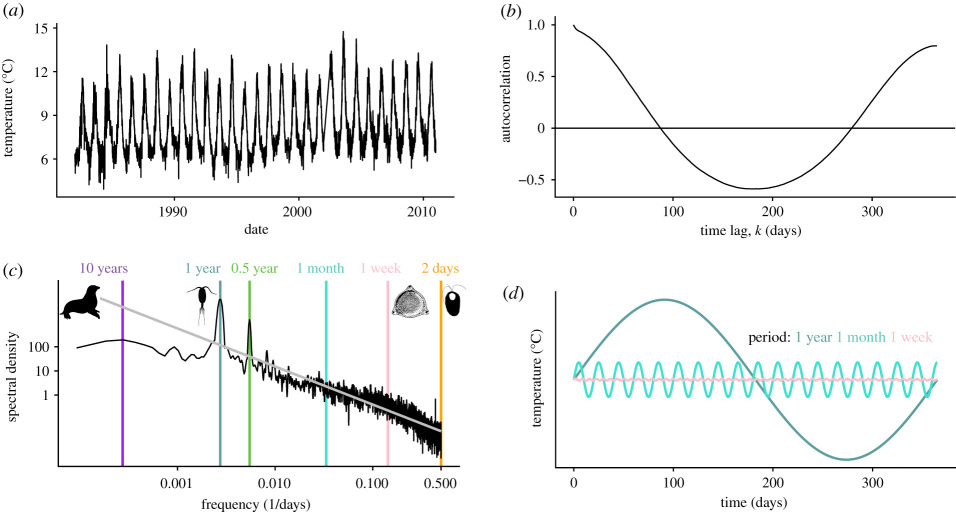

Box 1 figure 1. (*a*) Variation in daily sea surface temperatures at a site off the coast of Norway over the time period from 1981 to 2011. (*b*) A correlogram of the time series in panel (*a*), showing the autocorrelation between time points as a function of time lag, *k* (days). (*c*) A Fourier transform can be used to transform the time series in panel (*a*) to a frequency spectrum, which illustrates how the variance (power) is spread across a range of frequencies. The negative slope of this frequency spectrum, *β*, is −1.58 (95% CI −1.60, −1.56), characteristic of ‘reddened’ time series in marine environments. Coexisting organisms, including a harbour seal, a copepod, a diatom and a green alga, with different lifespans, experience different components of the frequency spectrum. (*d*) The Fourier transform decomposes the time series into a set of sine waves, each with a characteristic frequency and amplitude. Three of these frequencies (1 year (dark green), 1 month (turquoise) and 1 week (pink)) from panel (*c*) are illustrated here.

Wavelet analysis is an extension of spectral analysis, and is localized [16,17], in the sense that instead of estimating the variance spectrum of the entire time series, it estimates the frequency at each point in the time series. It reveals changes in the variance spectrum through time and so is particularly useful for examining non-stationary time series in the context of climate change.

**2. Predictability emerges from the temporal context of correlated events**

Correlations between two environmental variables, their cross-correlation in time, provide an opportunity for organisms to predict and anticipate future environmental conditions. For example, consider an environment in which two variables, temperature and oxygen, are correlated (box 1, figure 2). As illustrated in box 1, figure 2, if *x* is a change in oxygen and *y* is a change in temperature, and if organisms are capable of internalizing the correlation between these two variables (i.e. employ an internal model), they can exploit the correlation to anticipate a vital change in the environment. For example, they can use an increase in temperature as a cue that is associated with an impending drop in oxygen and adjust their metabolism (i.e. switch from aerobic to anaerobic metabolic pathways) accordingly. In this way, even if a change in oxygen *per se* is relatively unpredictable, as long as organisms can detect a change in temperature, they can initiate a metabolic response in advance of the change in oxygen, thereby increasing their performance relative to individuals who wait to sense and respond to the change in oxygen.

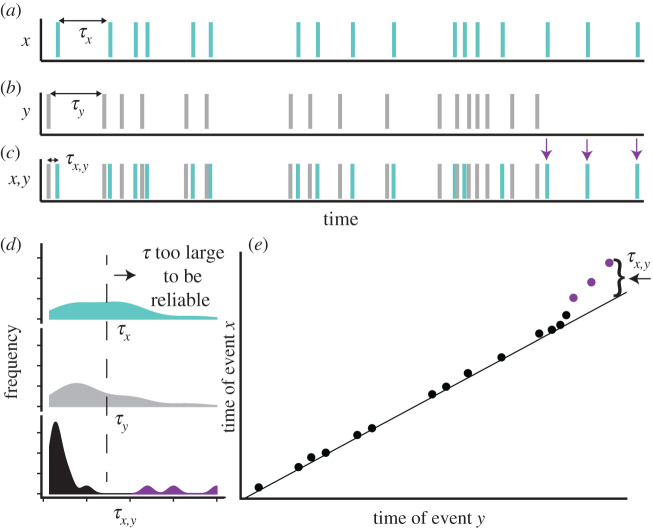

Box 1 figure 2. Organisms can exploit repeated associations between correlated environmental variables with a time lag to anticipate change. In this example, *x* is an event (i.e. a decrease in oxygen) that occurs in some random temporal sequence (*a*), as evidenced by the large variation in the time lags between successive *x* events, *τ_x_* (*a*,*d*, top panel). Similarly, *y* is another event, (i.e. an increase in temperature) that also occurs with a wide distribution of time lags, *τ_y_* (*b,d*, middle panel). In spite of the unpredictability of *x* and *y*, *x* is highly predictable within the temporal context of *y*, such that the delay between *y* and *x* is relatively constrained, as seen in the distribution of time lags between events *y* and *x*, *τ_x,y_* (*c,d*, bottom panel). If event *x* no longer occurs shortly after event *y* and the two event types become decoupled in time, as illustrated by the purple arrows in panel (*c*), purple dots in panel (*e*) and purple peaks in the bottom panel in (*d*), then the predictability of the environment decreases because the value of *y* as a cue for *x* decreases. Adapted from [18].

Box 2. **What is feedback versus feedforward, reactive versus proactive?**

Whether a process or event studied in ecology is reactive to the system's present state or proactive to an expected state is open to debate, but a clean and operational distinction can be made about what the organism (or any homeostatic system such as a cell or organ) senses and what information it uses to adjust its behaviour, physiology, etc., to the present, and likely future, environment.

In feedback control systems, the organism responds to a sensed or measured deviation in its *own* internal state, *z*(*t*), or performance relative to a desired, or reference, state (figure 2*a*, box 2 figure 1*a*). The organism senses a deviation and its distance from the desired state, regardless of what fluctuation causes this difference.

In feedforward control, the changes in the environment, *e*(*t*), are measured (e.g. cues, signals) and the organism's response is based on an internal model (box 2 figure 1*b*, figure 2*b*). In a strictly feedforward response, there is no feedback with self to assess a deviation from the desired state. It is the measured change in the environment, *e*(*t*), that causes the organism's behaviour or physiology to change. It is adaptive if the cue permits a response that maintains positive fitness under expected environmental change.

Indeed, in a feedforward system, the organism may simply respond to an external event and treat that event as a ‘cue’ (table 1) that is temporally correlated with other environmental conditions such that there is an order to them; one event can serve as a cue for a likely future event [24]. If that future event also presents a selective environment, then organisms that act on the cue to begin an activity such as development or migration may have a fitness advantage over others that do not. Certainly, more complex cognitive behaviours are also examples of feedforward systems, but cognition is not necessary, and there are many examples in which selection acts on responses to proximate cues that are correlated in time to future selective environments.

**Table 1.** Definitions of key terms.

term	definition	examples
living system	A self-sustaining biological system, characterized by flows of energy, materials and information processing. Synonyms: biological system, ecological system.	Cells, organisms, populations, symbioses, some communities.
cue	Environmental variable (either abiotic or biotic) that triggers an event or process and is predictive of a future environmental condition [26].	Variable features of the environment such as photoperiod, temperature, rainfall. For example, temperature is an environmental cue for sexual reproduction in many algal species, dispersal in fish or diapause in invertebrates. By sensing cues early in the season, organisms can anticipate the best time to initiate seasonal reproduction, migration, dormancy, etc., or to produce a particular seasonal morph, thereby matching their phenotypes to the expected conditions [27].
signal	Signals have four components [28]: (1) acts or structures produced by signalers, which (2) evolved for the purpose of conveying information to recipients, such that (3) the information elicits a response in recipients, and (4) the response results in fitness consequences that, on average, are positive for both the signaller and the recipient. By contrast to cues, which may contain information as a by-product of organisms' behaviour, signals have evolved for the specific purpose of conveying information and influencing others’ behaviour.	Pheromone trails laid by ants, peacocks' ornamented tail, electric pulses used by electric fish to communicate in water, bird songs.
prediction	A probabilistic conditional expectation about the future, informed by past and present events and an internal model. Allows organisms to prepare for impending changes in the environment [16]. ‘Prediction is not prescience but simply “output from an anticipatory model”’ [25].	Cells can internalize correlations between multiple environmental variables (e.g. temperature and oxygen), which allows them to express an appropriate energy-extracting metabolic pathway at the right time. Predictive behaviour is in contrast to stochastic switching, or diversified bet hedging, which allows for diverse phenotypes but does not require prediction of any particular future environmental state.
internal model	A simplified description of a system [25]. In organisms, this may be the physical instantiation of a probabilistic model [16]. We learn something new about a system by studying its internal model.	A model can be encoded in the pathways of a gene or metabolic regulatory network.
feedback homeostatic control	A process or mechanism whereby a system quantity can be returned to a constant level (the set-point), within a fluctuating environment. A deviation from the controlled set-point is countered by a controller that modifies the dynamics of the controlled system so as to diminish the error [29]. Homeostasis typically involves a negative feedback loop that counteracts the error. This type of control only responds to the state of the controlled system rather than that of the environment.	Thermoregulation in endotherms, food switching to achieve stoichiometric homeostasis (i.e. regulate elemental composition) [30,31].
feedforward homeostatic control	In a feedforward system, the control variable adjustment is not based on the self-state. Rather, the controller senses an environmental quantity, *e*(*t*), whose value is correlated to a likely future value of the state of the controlled system, *z*(*t* + *τ*). This introduces the role of prediction. The controller can modify the dynamics of *z*(*t*) according to the present value of *e*(*t*) *and* the state of *z*(*t*), so as to maintain constant the state of *z*(*t*). In feedforward control, disturbances are detected and accounted for before they have time to affect the system.	Negative phototropism, autumnal plant cessation of growth, immune priming, heat hardening, etc.
anticipatory system	To anticipate means to expect or predict. Rosen [24] defined an anticipatory system as a natural system that contains an internal predictive model of itself and of its environment, which allows it to change state in accord with the model's predictions pertaining to a later instant. In contrast to a reactive system, which can only react in the present to changes that have already occurred in the causal chain, an anticipatory system's present behaviour involves aspects of past, present and future.	An individual organism (an *Escherichia coli* cell, a tree, a copepod), any natural system that contains an internal model. See table 2.
phenotypic plasticity	Phenotypic plasticity refers to the ability of a single genotype to produce different phenotypes under different environmental conditions [27]. To do so organisms may use cues.	Plastic responses such as changes in development, behaviour and allocation of resources to competing demands can allow individuals to match their phenotypes (or those of their offspring, in the case of plastic maternal effects) to spatial or temporal variations in their abiotic and biotic environments. For phenotypic plasticity to be effective, organisms must be able to accurately forecast environmental challenges affecting their fitness.
colour of environmental noise (spectral colour)	Refers to the power spectrum of a stochastic environmental signal estimated by a Fourier analysis of the signal. By analogy to light, the colour refers to the profile of power across the signal's frequency spectrum [32].	Pink or red noise corresponds to variation that has more power at low frequencies; white noise is temporally uncorrelated and variance is spread equally across all frequencies [32,33].

**Table 2.** Examples of anticipatory mechanisms and internal models (correlations) on which they rely.

example	internal model
Circadian clocks in microbes, plants, mammals [71,72] allow organisms to time physiological processes.	Correlation between clock time and diurnal day/night cycle. Gene regulatory networks and metabolic pathways link the clock to particular biological processes, ensuring they peak at the appropriate times of day or night.
Toads sense water levels in temporary ponds, allowing them to switch to rapid metamorphosis [73] before ponds dry out.	Correlation between water level and time to pond drying.
Maternal light environment of understory forest herbs influences offspring life history and fitness, an example of anticipatory parental effects [74].	Correlation between maternal light environment and offspring light environment.
Reaching a critical short photoperiod is a cue used by boreal and temperate trees to stop growing in the autumn [75–77].	Correlation between photoperiod and impending winter conditions.
Negative phototaxis and daily vertical migration in *Daphnia*, *Artemia* [78] and marine invertebrates (e.g. crab larvae, copepods) are cued by a change in light intensity, and allow invertebrates to avoid visual predators by swimming to darker areas [79,80].	Correlation between light intensity and predation risk.
*Daphnia* reared in the presence of a predator produce predator-resistant offspring [81].	Correlation between maternal kairomone environment and offspring predation risk.
Immune priming in plants allows increased resistance to pathogen infection following previous exposure [82].	Correlation between pathogen exposure and likelihood of repeated exposure.

Feedback control is *reactive* since it reacts to changes in its own state, while feedforward is *proactive* since it acts ahead of the organism's expected change based on the environment's measured state. Feedforward systems also react to deviations, but they are in the measured state of the environment. In feedforward control, the system's output can change without any observable deviation from the desired state. While many types of organismal and system behaviours combine feedback and feedforward mechanisms, distinguishing these components is useful because it allows for a more mechanistic understanding of how these systems respond to environmental change.

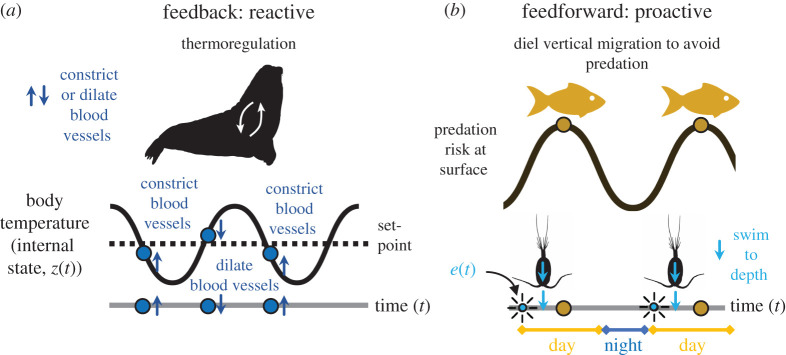

Box 2 figure 1. (*a*) Feedback processes are reactive and respond to changes after internal conditions have deviated from a set-point. In this example of thermoregulation, feedback control regulates the control variable, in this case, body temperature, by responding to the change in the internal state (body temperature, *z*(*t*)) of the organism. Blood vessels constrict or dilate (dark blue arrows) to cause the internal body temperature to return to the set-point after body temperature has dropped below or risen above the set-point temperature (points in time illustrated with blue circles). Note that blood vessel constriction or dilation (blue arrows) occurs after deviations from the set-point (dark blue circles). (*b*) Feedforward processes are proactive. In this example of diel vertical migration, descending to deeper waters at sunrise (light blue arrows) is a proactive response to light as a cue (external environmental state, *e*(*t*), light blue suns) to reduce exposure during periods of predation risk in surface waters (gold circles). Note that the copepods descend (light blue arrows) in response to the light cue (light blue suns), which precedes periods when predation risk is high (gold circles). By allowing systems to act proactively, feedforward processes avoid the delays inherent in reactive feedback processes. See figure 2 for an illustration of how copepods employ a combination of feedforward and feedback processes to avoid predation.

Reliance on feedback mechanisms to persist in fluctuating environments can be problematic. When organisms respond directly to their internal states, the time-delayed response makes them vulnerable to large and rapid deviations in their state that could cause death, and make their populations vulnerable to extinction. Fluctuating environments can cause regularly occurring stressful or otherwise poor conditions (box 1). Organisms or populations may perform better and be more likely to persist if they can minimize their exposure to these conditions or be phenotypically prepared for expected changes before they occur. To achieve this, living systems require processes that allow them to acquire information about the future state of the environment.

Many living systems persist in fluctuating environments by anticipating change through a variety of ecological and evolutionary cues and signal-based mechanisms (table 1, figures 1*d* and 2*b*, box 2, figure 1*b*). These mechanisms convey information about correlations between the state of the environment now and its likely state in the near future. These are *feedforward* mechanisms (table 1), in contrast to *feedback* mechanisms that rely on external cues and allow organisms to anticipate, prepare or prime themselves and/or their offspring for environmental change. Organisms use information acquired from their environment in many ways, and feedforward processes are pervasive in living systems. Feedforward processes allow organisms to buffer or exploit expected environmental change in a way that can enhance their fitness (figure 2*b*, box 2), and thus exist primarily as evolutionary adaptations. Circadian rhythms [34], phenology, phenotypic and behavioural plasticity [35] and transgenerational parental effects are all widely studied examples of feedforward mechanisms, even though they typically have not been classified in this way. Ecological and evolutionary models point to the long-term fitness benefits of feedforward processes [27].

While feedbacks are widely known to increase the persistence of living systems in changing environments, feedforwards are less well understood as a general class of mechanisms enabling persistence in fluctuating environments. Feedback and feedforward mechanisms differ in that feedback mechanisms are *reactive* processes that allow living systems to respond to environmental changes after they have occurred, while feedforward mechanisms are *proactive* and allow for living systems to anticipate changes in the environment before they have occurred (figures 1 and 2; box 2). While many biological processes and behaviours, such as behavioural thermoregulation or predator avoidance, combine feedback and feedforward components, it is useful to distinguish these components because the feedback and feedforward components may respond differently to environmental change, resulting in distinct outcomes for fitness and long-term persistence.

Here we review feedback and feedforward processes, drawing on concepts from engineering, systems biology, physiology, ecology and evolutionary biology, and convey their essential role in the adaptive responses of living systems in which many organisms are responding to variable and uncertain environments. We consider a generalized framework for feedback and feedforward processes, and demonstrate how feedbacks and feedforwards occur (or might occur) at the level of individuals [36,37], populations [10,38], and higher levels of organization such as communities [39,40]. We combine knowledge of how the biophysical environment is changing and how organisms, populations and communities can respond and adapt to change at different temporal scales. We argue that a framework combining feedback and feedforward mechanisms is required to achieve a robust understanding of how living systems persist in fluctuating environments and may be adapting to ongoing shifts in the structure of environmental fluctuations.

### (a) Characterizing correlations in fluctuating environments at different spatial and temporal scales

Fluctuations in physical, resource and biological conditions are a core feature of most environments. Here we address two features of this variability: (1) the correlation in time within single variables (i.e. autocorrelation) and (2) the correlations that exist among multiple variables (i.e. cross-correlations). Both auto- and cross-correlation patterns occur at the full range of scales and resolutions of space and time, and ecological systems reflect these features of temporal structure at more than one—but not all—scales. In box 1, we summarize methods we can use to quantify relevant scales of correlation, and we address types of correlations that allow organisms to time life events and behaviours that have consequences for fitness.

#### (i) Autocorrelation and predictability

Periodic, or repeating, temporal fluctuations occur at multiple scales and include diurnal and seasonal cycles of light and temperature, quasi-periodic variation in climates at multiannual (e.g. North Atlantic Oscillation, El Nino Southern Oscillation), decadal and millennial time scales (e.g. Milankovich cycles) (figure 1*a*). Aperiodic fluctuations also characterize variation in biotic conditions that link to niche relations, such as resource availability or predation pressure.

Characteristic features of fluctuations (i.e. predictability of environmental changes and periodicity of cycles) hold information that may be used by organisms to time important life-history activities to align with conditions best for survival, reproduction and growth [41]. Box 1 describes how we can characterize *predictability* of temporal dynamics in a single environmental variable, such as temperature. Predictability emerges when environmental variables are temporally or spatially autocorrelated, reflecting the increased likelihood that current conditions predict near-future conditions, such as long runs of above- or below-average conditions. Environments that are dominated by variation at low frequencies (i.e. cycles with long periods, high temporal autocorrelation; box 1, figure 1) are more predictable to organisms living in them because current conditions are likely to be accurate predictors of near-term future conditions.

#### (ii) Correlations among different environmental variables

Changes in environmental variables such as light intensity, photoperiod or rainfall that are correlated with some later selective environment can be used as ‘cues’ (table 1). Organisms use the information represented by cues in fitness-defining ways (i.e. timing of growth and reproduction). For example, a cue early in a season can allow organisms to anticipate future favourable conditions for reproduction, migration or development, and initiate the biological processes that will allow these life-history events to occur at the time of favourable conditions. In this way, organisms can match their phenotype to expected environmental conditions, increasing their fitness [27]. The value of a cue is related to the correlation, or mutual information [42], between a cue and a later environmental state. In other words, the benefit of the cue to an organism increases as the cue reduces uncertainty about the future environmental state.

#### (iii) Organisms experience the same environment differently

Species interact with the environment over a certain range of variation and not others, and this influences how they respond to and exploit temporal variation. Species may only detect and respond to fluctuations and cues at a given scale (box 1, figure 1*c*). Species with life spans on the order of a few years have typically evolved to coordinate key life events such as reproduction or hibernation with seasonal shifts in food, mate or predator availability. More generally, if organisms' generation times and lifespans are longer relative to the period of fluctuations, and individuals experience predictable environmental conditions, then feedforward mechanisms are likely to evolve [43,44]. By contrast, organisms occurring in environments that exhibit little variation within their lifetime, or highly unpredictable variation, are not likely to rely on cues and anticipatory mechanisms (e.g. [45]).

An environmental event or change in state that is used as a cue for one species may be noise for another species. When an environmental state, or fluctuations in that state, becomes used as a cue, the way this manifests depends on the life history of the species (box 1, figure 1). For example, frequencies of environmental variation that are detectable to an organism, and that are associated with variation in resources or other selective conditions, depend on the body size, life span and generation time, and these traits themselves are often highly correlated. Body size and generation time influence the frequencies of environmental fluctuations to which organisms may respond (referred to as ‘characteristic response times’, [46,47]) and the physical environment that organisms experience [48]. For example, a barnacle anchored to a rock in the intertidal zone experiences strong covariation in temperature, light intensity and oxygen availability over the course of a day. The same change in temperature and oxygen that was vital to the barnacle may be considered ‘noise’ to a fish swimming by.

The range of anticipatory mechanisms available to organisms depends on their capacity to acquire and respond to information about their environment and their current state [49]. Sensory systems allow organisms to detect both their state and the state of their environment. Sensory systems differ among species, and can even vary among individuals within populations and also among developmental stages [50]. Different sensory modalities (temperature, vibrations, electromagnetic energy, chemicals, etc.) and sensory systems (vision, hearing, electric field detection) allow organisms to detect different types of cues. The types of sensory stimuli that an organism is able to detect may determine its ability to find food, compete for resources [51–53] and avoid predators [53,54]. In the presence of ubiquitous background noise, species differ in their sensory systems and abilities to separate signal from noise, so the same environment is experienced differently by different species. As with other life-history traits such as size or generation time, sensory systems may have evolved in some cases in the context of feedback and feedforward processes in varying environments.

## 2. Integrating concepts from systems biology to classify strategies for dealing with fluctuating environments

Living systems are characterized by their capacity for homeostatic control, which is the capacity to maintain a viable state, despite variability in their environment. A homeostat is any set of processes or mechanisms that results in a system property or process being maintained at a (quasi) constant level, within a fluctuating environment. Variables held under homeostatic control remain within a narrower range of values than if they were not regulated; the regulated value typically occurs within a range that is consistent with the viability of the organism or system.

Here we describe how homeostasis is achieved via feedback and feedforward control mechanisms (figure 2; box 2, figure 1). We will see that feedback and feedforward processes are integral to a general approach to homeostasis and the persistence of organisms and other living systems in fluctuating environments. This classification expands on an earlier framework proposed by Rosen [24,55]. Feedback and feedforward systems (including model-predictive control) have been the focus of a great deal of research in complex system science, engineering and theoretical biology (e.g. [56,57]). Like any classification, this is just one way of understanding how feedback and feedforward processes have shaped systems to respond to fluctuating environments, and it is meant as a framework to locate the focus of future analysis, to guide inquiry about change in ecological systems and to facilitate comparisons among systems.

## 3. Class 1: feedback homeostats

Homeostasis by negative feedback is the most familiar form of adaptation in physiology [58]. The mechanisms, such as regulatory pathways, leading to homeostasis in body temperature, water content, energy levels, nutrients and essential cofactors (e.g. iron) are well studied in a host of model and non-model organisms [59]. For example, in one-third of the oceans, the bioavailability of iron limits primary production, and phytoplankton have evolved strategies to acquire and recycle iron even when it is extremely limiting. For example, the picoalga *Ostreococcus* uses the protein ferritin to regulate iron uptake and recycling, and this iron homeostasis is essential for cell survival under iron limitation [60].

In abstract terms, any homeostatic system can usually be decomposed into a controlled system or process (some aspect of an organism's physiological system) and a controller (e.g. a regulatory pathway; figure 2*a*). In reality, there may be no simple dichotomy in these subsystems, but in many cases one can identify processes that fall into each. The key property of feedback homeostatic systems is that the receptor (i.e. sensor) only measures the *internal* state of the controlled system, *z*(*t*), and not the environment (figure 2*a* versus figure 2*b* and box 2). Deviations of the state of the controlled system away from the homeostatic state result in a response modifying the dynamics of the controlled system so as to diminish the deviation from the set-point (negative feedback). In the simplest cases, homeostats have no memory of past states. An example of this is the thermostat controlling the temperature of your room, which functions by controlling the actions of a heating system based on deviations in temperature from the given set-point. In endotherms, thermoregulation occurs when the cooling of the blood is detected by receptors and stimulates centres in the brain (controller), which ‘turn on’ heat-producing mechanisms of the body (effectors) and the body temperature is adjusted back to the set-point so that temperature is maintained at a constant level [29] (box 2, figure 1*a*).

Feedback mechanisms allow populations to adapt to fluctuations in their environment, reducing the variation in their internal physiological state. In addition to feedback mechanisms that operate by adjusting physiological conditions internally, organisms may also use behaviours that allow them to avoid high-frequency and potentially damaging environmental states [61,62]. For example, intertidal organisms exposed to high temperatures and desiccation stress at low tide can buffer their exposure to thermal fluctuations by becoming inactive during exposure extremes (many animals cycle between activity and inactivity on a daily basis) [63]. Organisms with a broader range of thermal microenvironments have greater opportunities to thermoregulate, and access to these microenvironments depends on motility, body size and features of the environment. The combination of behavioural thermoregulation and controlling activity patterns allows organisms to avoid variation in body temperature, especially at daily and annual frequencies [61,64]. Notably, these feedback mechanisms do not require internal models that relate events separated temporally, just the ability to sense the internal state and respond as feedback homeostats.

Feedback homeostats function as a result of variation in their environment and allow organisms to maintain steady-state conditions in a range of vital processes in fluctuating environments. The aggregate response of many individuals forming a population reveals variation among individuals in their capacity to maintain homeostasis in a dynamic biotic and abiotic environment. The performance of feedback mechanisms varies in their responses to deviations from steady state, which is arguably why this topic has been the focus of so much theoretical research in ecology and evolution. The set-point or long-term steady state around which feedback regulation occurs is often variable and may be under selection. Species vary in their capacity to achieve homeostasis under limiting or stressful conditions, so competition among genotypes within and among species is key to understanding the diversity of homeostatic strategies, and the overall functioning of populations and communities under novel patterns of environmental change.

Feedback mechanisms can be adaptive, in the evolutionary sense, when they involve behavioural or physiological traits with a heritable genetic basis that increase fitness. An example of such an adaptive response might be when a lizard responds to a warm body temperature by moving into the shade in genetically encoded adaptive behaviour that improves fitness [65]. Feedback mechanisms may also occur even if not directly underpinned by heritable gene systems, and thereby be ‘non-adaptive’ in the traditional evolutionary sense. For example, processes driven by physical constraints and dynamical processes such as resource-limited population abundance and coexistence of competing species are feedback processes that, in and of themselves, are not under selection. At the community level, feedback processes may be dynamical consequences (e.g. stability) arising from altered birth and death rates owing to the effects of another species, such as predator-mediated density-dependence. These higher-level feedback processes may contribute to the persistence of a system. Here we consider feedbacks within organisms that are adaptive in the evolutionary sense [66], as well as feedbacks that operate at higher levels of biological organization (populations, communities and ecosystems), that contribute to the persistence of living systems [67–70] in fluctuating environments. While distinguishing between feedbacks that arise via natural selection versus those occur owing to other mechanisms (e.g. physical constraints) is important to understanding how they may change as the environment changes, considering feedbacks in multiple forms allows us to understand the processes that affect persistence of living systems at multiple levels of organization, from cells to ecosystems.

## 4. Class 2: feedforward homeostats

Feedforward homeostats add the capacity of the controller to measure the state of the environment. We continue to use the language of systems science to refer to the components of the system that integrate the sensed information from the environment and the consequence for the focal system. The controller may be a nervous system, as in vertebrates, but the term can be applied much more broadly to any part of a network that relates signal and response. In feedforward systems, a controller can sense an environmental quantity (via the receptor) whose present value *e*(*t*) has historically—in the experience of the controller—been correlated with a likely subsequent value of the internal state (*z*(*t* + *τ*)) of the controlled system (figure 2*b*). The temporal correlation between *e*(*t*) and *z*(*t* + *τ*) is modelled by the controller. In feedforward homeostats, the controller can modify the state of the controlled system in accordance with the present value of *e* and *z*, so as to keep constant some required function of *z*. Feedforward mechanisms differ fundamentally from feedback mechanisms because the system is using information about the environment (e.g. cues) to predict and prepare for a later state. The correlation between *e*(*t*) and *z*(*t*+*τ*) represents a model (in an abstract sense) that has evolved in a system in which environments at one time and internal states at another have been historically correlated. For this reason, they are sometimes classified as anticipatory systems [24] (table 1). The internal model must encode the range of environmental conditions to which the controlled system has historically (evolutionarily) been exposed and is expected to encounter. In cases where the feedforward system's model does not accurately predict *z,* perhaps because the historical temporal pattern in the environment is no longer occurring, or the environment now includes new states, then the feedforward response will no longer benefit the system. If this situation becomes common and is chronic, then it is no longer beneficial and may be maladaptive, as it will threaten the viability of the organism and the population if the maladaptive state occurs for several generations.

Feedforward mechanisms have some advantages over feedback mechanisms. The controller response is no longer purely deviation- or error-driven, meaning that the internal state need not deviate or degrade before it responds. Any purely feedback homeostat has an intrinsic time delay (constant) so it risks failure before a corrective response can be activated. In environments that fluctuate rapidly, or in novel ways, a feedback control system will track the fluctuations rather than exhibit steady state, or homeostasis. Feedforward control systems operate based on regularities in the environment (the correlation between *e*(*t*) and *z*(*t*+*τ*); box 1, figure 2), rather than off the deviations around the set-point, or steady state of *z*, that the feedback mechanisms use. By adjusting ahead of the environmental change, feedforward mechanisms avoid the costs of constant error correction. The key distinction between feedback and feedforward mechanisms is that while feedback mechanisms are *reactive* and rely on internal deviations from a set-point, feedforward mechanisms are *proactive* and add the use of cues from the external environment to maintain a set-point (box 2). We note that in nature, feedback mechanisms can occur through a variety of biological processes over different time scales. These processes include adaptation by natural selection and population dynamic processes under physical constraints (e.g. population- and community-level negative feedbacks leading to stability), while feedforward mechanisms could arguably only arise in a system that has evolved the ability to measure and anticipate the state of the environment in order to persist in a variable environment.

Examples of feedforward control are very common in biology (table 2). Any behaviour or activity that uses a cue to prompt its timing is predictive and model-based. Major examples are most forms of phenotypic plasticity, and adjustment of organism timing. Many organisms, ranging from single-celled algae to mammals, use circadian clocks (a type of internal model) to anticipate regular environmental changes and coordinate internal biological processes [71]. For example, plants upregulate photosynthetic machinery before dawn, allowing an immediate response to light when the sun rises [83]. The importance of these anticipatory mechanisms is demonstrated by the fact that when circadian clocks are disrupted, fitness decreases [84,85]. Plants and animals prepare life histories in spring and winter on the basis of day length rather than internal temperatures. For example, trees stop growing and shed their leaves in autumn based on day length cues in anticipation of impending winter [86]. Anticipatory developmental switches between alternative phenotypes (i.e. direct development and diapause) are often cued by photoperiod and have evolved independently in a wide variety of taxa [43,87,88]. These switches are often established and maintained if cues are reliable (i.e. they are accurately correlated with later fitness-defining environmental conditions) and available to the organism at the appropriate time to influence development.

Feedforward mechanisms can also operate across generations. Parents can modify the phenotype of their offspring in response to changes in the environment that act to increase parental fitness by also increasing offspring fitness [89], using a set of mechanisms called anticipatory parental effects, which are a type of transgenerational phenotypic plasticity. Anticipatory parental effects are expected to occur in situations where parents can detect and identify current environments, parental environments accurately predict offspring environments (i.e. the cues are reliable) and parents can accurately transmit information to offspring so that it can be integrated into offspring phenotypes [90–92]. By contrast, populations that experience completely unpredictable and variable environments are not likely to evolve anticipatory parental effects [42,93]. If environments are variable and unpredictable, then diversified bet hedging, in which parents produce offspring with a variety of phenotypes, may be a better strategy [38].

Dormancy is a common feedforward strategy to enable persistence in variable environments [94–96]. Dormancy in plant seeds allows seeds to avoid germination during periods that are only temporarily favourable, and dormancy can distribute offspring over time and bet-hedge against unpredictable variable environments [97,98]. Dormancy and germination cueing can allow populations to colonize new locations and persist in changing environments by ensuring that germination occurs when environmental conditions are appropriate, and to escape from crowding and competition [99,100].

Feedforward systems are expected to arise when the environment varies in a highly regular pattern for a long period of time. Feedforward systems may not be robust or attuned to variation regimes that have no historical precedent and are therefore not modelled by the system. If some properties of the environmental fluctuations change so that the system's internal model is no longer accurate or predictive of the future internal state, then, in evolutionary terms, the model is maladapted, and fitness may decline. But the advantage is that, under conditions with a long historical precedent, systems with feedforward processes are prepared for their likely future. Phenological life-history responses such as when trees time spring flowering in response to temporal patterns of temperature in the autumn and winter represent a feedforward process to allow maximum growth and reproduction of trees in seasonal climates. However, as climate changes and the correlation between day length and temperature shifts, the timing of flowering may shift and may not be as well aligned with other springtime events as in the past [101]. When interacting species rely on different cues, and these cues change at different rates, this can lead to trophic mismatches [102].

## 5. Class 3: general adaptive systems

General adaptive systems (GAS) are characterized by combined feedback and feedforward processes [29,55,103,104]. GAS integrate measures of multiple environmental states and can develop multiple models linking their internal state to different *e*(*t*). They can also modify their internal models *and* features of their environments in order to achieve desired future states. GAS can acquire the ability to measure and integrate different sensory modalities about the environment's states; these can include a mix of visual, audible and olfactory states of the environment. Over extended periods of time these multi-modal models of the environment may improve an organism's expectation of its fitness and therefore allow a more adaptive short- and long-term response to fluctuating conditions. This feature boils down to an individual being able to learn and acquire new sources of information from the environment to reduce uncertainty in the measurement of its state (epistemic uncertainty), and to more reliably anticipate its performance under fluctuating conditions.

Theory predicts that learning (i.e. updating of internal models) should be favoured when the environment is variable and organisms can get reliable cues, and this has been supported empirically in a range of taxa [105]. Learning allows individuals to anticipate and adjust in advance of events with major physiological impacts. For example, physiological pre-adjustments mediated by learning can increase tolerance to extreme temperatures [106], male reproductive success and predator avoidance [107]. In great and blue tits, *Cyanistes caeruleus* and *Parus major*, respectively, matching the timing of nestling feeding with the local peak in food abundance increases fitness [108]. Since peak food abundance varies among habitats, birds must predict the peak a few weeks in advance to time their egg-laying appropriately. They rely on photoperiod cues [109], but can also alter timing of egg-laying based on experience with previous breeding seasons [110].

The ultimate adaptive ability of a GAS is the development of a set of behaviours to modify and manipulate the state of the environment, *e*, using some sort of effector. Modification of the environment is directed so that the environmental conditions permit the system's future state *z*(*t* + *τ*) to more closely match its physiological requirements. This capacity to control the state of the environment falls into definitions of ecosystem engineering [111,112].

In the context of global environmental change where anomalous patterns of environmental variation are occurring with increasing frequency, it is not clear whether adequate evolutionary potential exists in existing feedback and feedforward mechanisms to ensure long-term persistence of some living systems. The extent to which historically calibrated feedback and feedforward systems will allow living systems to persist in the future on our changing planet will depend on the type of environmental variability organisms experience compared to what they experienced in their history, which we discuss below, and may be altered under global environmental change.

Ultimately, there are always limits to the predictability of natural environments. All feedforward mechanisms are limited by the internal model *and* the fundamental limits to predictability (ontological uncertainty—uncertainty of future external and internal states). The degree to which feedback and feedforward processes are critical to the existence and persistence of biological systems likely depends on the predictability of the environmental fluctuations and the relative costs and benefits of anticipating versus reacting to environmental changes. Maintaining an internal model that is required to anticipate future internal states can be costly, and the degree to which organisms use feedforward mechanisms depends on the costs and benefits of anticipatory behaviours [113,114]. For example, sensing mechanisms involved in chemotaxis have a metabolic cost, and presumably the cost increases as the accuracy of sensing increases [115]. Learning and memory may entail fitness costs owing to the energy and materials required to acquire and store information [116]. Unavoidable delays between measurement and response involved in feedback strategies also induce a metabolic or fitness cost. The fitness benefits of feedforward mechanisms are related to the degree to which the ability to detect and act on cues improves expected fitness of the offspring [42,117]. Ultimately, there may be a fitness trade-off between responding late (i.e. simply reacting and not anticipating) and the fitness cost of maintaining highly accurate sensing mechanisms.

## 6. Evidence for feedback and feedforward processes in ecological systems at higher levels of organization

Ecological systems are hierarchical in nature, and different levels of the hierarchy are defined by feedback processes. Populations are ecological units defined by the genetic processes of reproduction at the population level; communities can be defined as the number and diversity of species in a defined space or time, in which richness often remains stable in dynamic equilibrium while population dynamics operate at the level of populations [118]. Ecosystems have long been recognized as ecological systems defined by energy and material cycling, and even information processing, in which feedbacks operate to determine ecosystem structure and stability. There are two ways to consider feedback and feedforward processes at higher levels of organization. The first is to focus on how feedback and feedforward mechanisms within individuals and populations ‘scale up’ to influence higher-order ecological processes, and the second is to consider how they operate independently at those higher levels of biological organization. This second approach recognizes functional levels of organization beyond the population, and has a robust history in the fields of ecosystem and systems ecology employing general concepts of feedback, feedforward and information processing [119–121]. In this view, the collective dynamics of populations and entire assemblages of species can be analysed and understood from the point of view that all living systems are exploiting the information in variable, autocorrelated and cross-correlated environmental conditions, enabling them to persist in fluctuating environments.

### (a) Population level

A major challenge is to understand how information used by individuals to adjust their behaviour, movement, aggregation and reproductive investment through feedback and feedforward mechanisms scales up to mediate population fitness and dynamics when environments vary in their quality and predictability over time [122]. Population models differ in the way they formalize fine-scale variation about individuals (e.g. phenotypic traits, life histories and behaviours) and at what level conditional information about the state of the environment is used by individuals. Decisions about how to model these features can have strong effects on resulting population dynamics and the predictions these models make about the effects of changes in environmental variation [122–125].

Autocorrelated environmental fluctuations are currently understood to have large effects on the mean and variance of population dynamics and on the probability of extinction and colonization. The evidence stems from a large body of theory for unstructured [126–130] and stage-structured population models [131,132]. This has been supported in laboratory experiments [10,133,134] and analyses of large databases of population time series [135].

Temporal autocorrelation in environmental conditions is expected to have interactive effects with population size when density-dependent processes are at play, such as resource-limited growth, such that time-integration of the environment is not simply additive [12,129,136]. Autocorrelated variation can also mediate the timing of switches when multiple population equilibria exist [127,137], which defines their resilience. Thus, the population model is a linear or nonlinear filter of the environment where the feedbacks (e.g. density dependence, or switches in equilibria) are predicted to either dampen or amplify the stochastic environmental signal, with predicted impacts on population extinctions risks [137–139]. Depending on how they are modelled, feedbacks arising from density dependence can have a strong effect on the variance and extinction risk of the populations [137].

Simple population models often involve no time delay between the environment and the population response. Relaxing the constraint that all events happen instantaneously can greatly alter how density dependence is expressed in population dynamics [125]. An important class of models exists that integrates time delays in the model to reflect how vital rates observed in the population arise from previous historical environmental states (e.g. because of changing seasons). Among these are models that incorporate feedforward response systems such as when the environments experienced by parents can mediate the phenotypes and fitness of their offspring [140–142]. In general, encoding these intergenerational effects into population models can produce distinct and complex dynamics [125,140]. For example, maternal effects generally increase population variability in these models [140].

In general, when there is temporal autocorrelation, current conditions not only determine the consequences of current decisions that individuals make, but they are also informative of future conditions [143]. Population theory suggests that accounting for anticipatory parental effects and phenotypic plasticity is important and is improving our understanding of population-level outcomes of changing environmental conditions. Changes to environmental predictability of any form (see box 1) in either the abiotic and biotic environment may lead to maladapted cues. The demographic consequences of these fitness declines and the extent to which evolutionary or plastic changes in cue responses can promote recovery are generally unknown.

## 7. Community level

Ecological communities are ensembles of species whose populations interact through dynamic processes such as competition, facilitation and predation. Community-level patterns can reflect these interactions [144] and can also reflect constraints at the community level that are not driven by the dynamics of any particular species [145]. Considering feedback and feedforward processes at the community level, we may take the first approach of considering how feedforward mechanisms within individuals influence species interactions. When species strongly interact, fluctuations in abundance of one species can cause the other to respond, hence a varying environment may be both abiotic and biotic. The abundances of Canada lynx and snowshoe hare fluctuate in iconic predator–prey population cycles, out of phase such that peak lynx abundance is followed by very low hare population sizes. These cycles have persisted for centuries, well documented by fur trapping records [21]. Initially, resource limitation was thought to be the primary driver of hare population cycling, which then was assumed to cause declines in lynx abundance, reflecting food limitation. However, resource limitation could never fully explain the cycles. Now, the explanation includes processes based on feedforward mechanisms in the form of maternal effects [146]. The first of these is that hares experience physiological stress when lynx abundances are high and predation rates are high. Stressed mother hares are less successful at reproduction and pass on symptoms of stress to their offspring. It has been hypothesized that maternal stress and subsequent risk-sensitive behaviour in young hares may be a form of maternal adaptive programming [146]. Juvenile hares with higher stress hormone levels spend more time under cover and are less active during field trials, highlighting a potential mechanistic route to allow individuals to cope with a changing environmental risk of predation [147]. A second possible feedforward process occurs in lynx. Research on lynx in Newfoundland, Canada, suggests that when prey are scarce, daughter lynx remain in their mother's territory, repressing their own reproduction during times of hare shortages. Repression of reproduction keeps densities low and allows the same individuals the chance to reproduce in a subsequent year when hares may be more abundant [148]. This picture of the role of feedforward mechanisms at the individual and population levels is based on reciprocal, density-dependent species interactions, and this system of interactions allows population-level feedforward and feedback processes to propagate to the community level, because these two species play important roles in their community.

### (a) Cue-based synchrony in reproduction and species persistence

In environments that do not experience large environmental fluctuations in light or temperature, some species have evolved the use of complex combinations of multiple cues to time life-history strategies and synchronize reproduction events. On coral reefs, the high biodiversity and low abundance of many species present challenges for reproduction and mate finding. Some species use a combination of light, temperature, lunar and diurnal cues to reproduce at specific times of year—only once per year, and within the same hour [149]. Conspecifics use the same lunar cues, increasing the probability that gametes from the same species will encounter each other and fertilization will occur [149]. These spawning events not only increase fertilization rates of rare species, but they also provide a pulse of food for consumers. The diverse, biological system associated with coral reefs in a relatively stable abiotic environment has generated its own fluctuations in the environment that have in turn become a selective environment for the timing of releasing gametes [149].

Co-occurring species perceive and respond to varying environments differently and these differences underpin explanations for the maintenance of diversity in competitive communities. For example, primary producers have evolved the use of different cues in the same environments; some species begin budburst and leaf-out earlier than others in the same locations [150]. These differences may reflect evolved partitioning of the temporal niche by primary producers. Phenological trade-offs between timing and productivity are at the heart of plant coexistence mechanisms [151]. Temporal storage effects are another important mechanism for coexistence of species in fluctuating environments [39,152,153]. For example, in aquatic systems, resting stages can be stored in sediments to emerge later, allowing species to ‘recolonize’ their environment rather than being lost when conditions are unfavourable [94], thereby maintaining biodiversity in the system. Similarly, the long-term coexistence of winter annual plants in the Sonoran desert is based on functional trade-offs in growth rates and low-resource tolerance [154]. Species separated along a trade-off between growth capacity and low-resource tolerance have different demographic responses to precipitation variation across years, leading to a different set of species present in any given year from a broader seedbank. In this case, early seasonal cues select for different species as the environment varies, maintaining higher diversity over time. Trade-offs in how species grow in fluctuating environments are increasingly understood to mediate community-level climate change responses [150,155] and biodiversity changes via the establishment of non-native species [156].

Differences among species in their internal models of the environment can also maintain diverse food webs. In temperate aquatic systems, many plankton populations shift from stationary overwintering growth phases to fast-growing phases when photoperiod becomes suitable and temperatures warm [157,158]. The spring bloom is the most intensely productive time of the year in many pelagic systems, in which much of the annual carbon is fixed before resource limitation sets in. The timing and magnitude of the spring bloom influence ecosystem structure and function for the following year. Shortly following the spring phytoplankton bloom and sudden resource availability, zooplankton populations grow rapidly, grazing down fast-growing phytoplankton populations. Young-of-year fish consume zooplankton, allowing fish to grow and spawn. The timing and magnitude of the bloom, and its importance, exist because of temporal (annual) fluctuations in light and temperature. The variety of biological processes that respond to this regular environmental fluctuation including phenological cues on daylength and temperature, with temperature triggering the end of diapause for some zooplankton populations, and onset of dormancy of other populations through the use of resting eggs or diapause stages [158,159], enable the maintenance of diversity in these communities.

### (b) Diversity and feedbacks at the community level

Feedbacks can also occur and maintain organization at the community level. Feedbacks at the community level include any process in which the output affects the input and tends to maintain a variable around a relatively constant state, enabling persistence [67,68]. Such feedbacks have been considered to underlie the finding that species richness at the community level is relatively stable even while environmental conditions and the composition of species can vary substantially over time [160–162]. Compensatory dynamics describe the negative correlation among species' abundances within the community—suggesting one compensates ecologically for the other, in a negative density-dependent manner [163]. When one species increases in abundance, others decline such that total diversity or energy flux remains consistent throughout the change. Hence, community functions may remain within certain bounds, enabling community persistence. When coexisting, competing species exhibit negative covariances in population dynamics, such that the total resource use at the community level remains more stable than would be expected by chance or by independent population changes not connected temporally through the interaction [161]. Compensatory dynamics are thus an example of increased stability via negative feedback at the community level in a varying environment. However, we are not arguing for a fixed set-point value for species’ diversity, rather that feedback and feedforward processes arising from interspecific interactions for limiting resources tend to balance extinction and colonization, keeping diversity within bounds. Over the very long-term (i.e. paleoecological scales), variation in environmental constraints linked to climate and resource availability will mediate non-stationary variation in biodiversity [164].

When communities act as collectives, feedback and feedforward processes may operate together to affect patterns and processes at the community level. For example, chemical communication in bacteria in the form of quorum sensing occurs in response to changing conditions in the environment, such as a high cell density. Quorum sensing causes collective gene expression and behaviour, involving feedforward and feedback regulatory loops that rely on the production and detection of extracellular signalling molecules (autoinducers) [40]. The internal models that enable feedforward processes at the community level are contained in the architecture of quorum sensing networks, and bacterial communities can tune their input–output relations to changing conditions, enabling them to operate as GAS. Quorum sensing can result in the formation of mixed-species biofilms with an array of competitive or cooperative interactions [165–167]. Other examples of feedforward processes operating via quorum sensing at the community level include the cues that induce bioluminescence in multispecies assemblages of microbes [168] and shared information that leads to pathogen resistance in microbial communities [169]. Collective behaviours and group-decision making are not limited to bacteria, they are common in eukaryotes (e.g. yeasts), and may arise between kingdoms (i.e. between bacteria and their metazoan hosts) across the entire Tree of Life [170].

## 8. Anthropogenic influences on environmental fluctuations

There is clear evidence that humans are changing the way the environment fluctuates [33,171]. Several key statistics, such as the variance, autocorrelation and periodicity of environmental fluctuations, are predicted to change over the coming century [8,9,172]. Humans are also altering the reliability of the correlations underlying many environmental cues as their timing and phases shift over time, within and across years. There is also evidence that humans are modifying the ability of organisms to detect cues [171,173,174]. Changes to the sensory environment, such as changes in light and acoustic conditions, visual properties of water or additions of chemical compounds may distort the production, transmission and perception of signals and cues. For example, metal and chemical pollutants influence the development and production of signals by influencing endocrine function and other cellular processes involved in signal production [175]. We now assess the evidence for human-induced changes in (a,b) cue reliability and detectability and (c) the temporal structure of environmental variability.

### (a) Changes in cue detectability

Human impacts on ecosystems are distorting or altering auditory, visual and chemical cues and hampering focal organisms' ability to detect them [173,176,177]. Acoustic pollution from human sources interferes with the detection and discrimination of acoustic signals. For example, low-frequency, human-generated noises in aquatic ecosystems, such as noise from boat traffic, often overlap in frequency with the hearing range of most animals and the frequencies of the calls of many species, including marine mammals [178]. By masking acoustic signals, humans are effectively decreasing the distance from which an individual is able to detect a conspecific's call and making auditory cues more difficult to detect. Human impacts are also altering the visual environment. Eutrophication and run-off are altering the availability of light in aquatic environments, and changes to the bandwidth of available light can have severe consequences for the detectability of cues among aquatic species. Eutrophication in Lake Victoria has altered the light environment such that two species of cichlid fish have hybridized because females are unable to distinguish red males from blue males [179]. High turbidity levels reduce the distance from which predators can see their prey, which reduces foraging efficiency and food intake in brown trout [180] and Eurasian perch [181]. Artificial light sources associated with human settlements and ships on the ocean are altering lightscapes. For example, when artificial lights are brighter than the horizon over the ocean, sea turtle hatchlings move towards human settlements instead of the ocean [182]. Together, human-induced changes in the sensory environment influence organismal fitness by altering individuals' ability to find food, avoid predation, acquire mates, provide parental care and interact with various aspects of the biotic and abiotic environment.

### (b) Changes in cue reliability

Many feedforward mechanisms rely on light as an information source, and artificial light pollution can cause adaptive feedforward mechanisms that rely on light as cue to become maladaptive. Many organisms use lightscapes as cues for directional movement [182], and changing lightscapes can result in disruptions to movement patterns. For example, nighttime light can alter nocturnal downstream migrations in Atlantic salmon [183]. Artificial light pollution influences the orientation of individuals that rely on visual cues for daily movement [184] and may disrupt light-cued diel vertical migrations in zooplankton [185,186]. Artificial light after dusk or before dawn can cause phase shifts in circadian rhythms, either by delaying or advancing the cycle relative to natural diurnal day–night cycles and thus cause physiological functions to become out of phase with relevant ecological conditions. Persistent levels of low light or short pulses of bright light from ships or cars can be enough to entrain circadian rhythms [187,188]. In addition, artificial light can lead to mistiming of events that require photoperiod cues. For example, some species of deciduous trees maintain their leaves for longer in autumn in the vicinity of street lights [189,190], potentially leaving them exposed to higher rates of frost damage.

**Figure 3.**
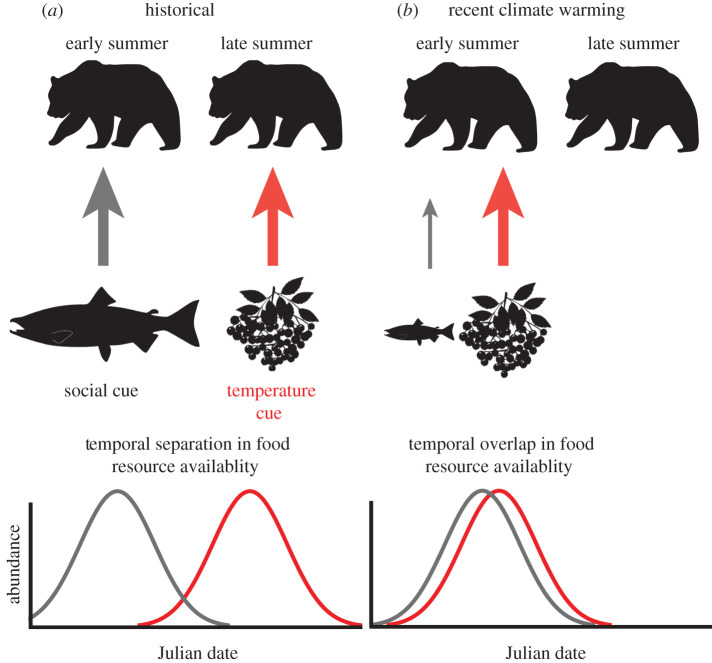
Variation in phenological cues used by salmon and elderberry alter pathways of energy flow in food webs as the climate warms. (*a*) Historically, brown bears fed on stream-spawning salmon and then switched to feeding on elderberries once they were ripe, later in the summer. This temporal separation in resource availability allowed bears to feed through an extended period of the growing season. (*b*) In recent years, red elderberries have begun ripening earlier in the summer while the salmon have continued spawning at the same time. This means that red elderberries are available to bears at the same time as the stream-spawning salmon. That the elderberries have altered their phenology more than higher trophic levels, including salmon and bears, may be common across ecosystems, since primary producers tend to be more sensitive to abiotic environmental cues [102]. The newly established synchrony in resource availability for bears may fundamentally alter energy pathways in this coastal ecosystem. Based on data from [197,198].

Disruptions in relationships between historically related conditions (i.e. cross-correlations between temperature and day length) may alter the outcome of species interactions. If individuals evolved to rely heavily on one correlated environmental cue, and that cue is no longer a good indicator of some physiologically relevant condition at a later time, then this may result in the mistiming of important life-history events and lead to phenological shifts [102,191–193]. In a community context, different organisms use different cues for their phenologies (i.e. temperature, rainfall, photoperiod). Phenological mismatches may occur across trophic levels when the cue used by one trophic level changes at a different rate to the cue used by a higher trophic level [192,194–196] (figure 3). Consumers generally have lower sensitivity to environmental cues than their resources and, as a result, they generally have weaker responses to changes in the cue than their resources, leading to potential mismatches in consumer–resource interactions [199–201]. Even if both interacting species use the same type of cue (e.g. temperature), these cues may occur at different times of the year or have different dimensions (e.g. duration, frequency, mean, extreme), and since temperatures at different times of the year have been shifting at different rates, phenological mismatch may occur [199]. This is also one possible explanation for high variation in species’ geographical range shifts [202]. Similarly, even the same cue, at the same time of year, can elicit different responses in co-occurring species [150].

In a food web context, differences in the cues used by different species to time life-history events can cause shifts in major energy channels as the climate changes. In coastal Alaskan ecosystems, brown bears feeding on salmon are a critical link between marine and terrestrial ecosystems. Typically, brown bears feed on stream-spawning salmon early in the summer, and then switch to feeding on elderberries later in the summer [197] (figure 3). The sequential timing of the arrival of stream-spawning salmon, followed by elderberries ripening creates a relatively long period of foraging opportunities for bears during the short Alaska growing season. As spring temperatures have warmed, elderberries have shifted to ripening earlier in the summer, overlapping more with the stream-spawning salmon. One potential explanation for the difference in relative phenology shifts is that salmon and elderberries rely on different environmental cues. While elderberry phenology is likely cued by temperature [197,203], the cues salmon use to time their migrations are likely a combination of temperature, stream flow and social information [198,204–206]. When both resources are available at the same time, bears prefer elderberries and abandon the salmon. This climate-induced diet-switching by the bears owing to synchronized resource availability may fundamentally alter energy flows in stream food webs. Bears feeding on stream-spawning salmon play a large role in modulating energy pathways in the food web and are capable of transferring large amounts of marine-derived nutrients into terrestrial ecosystems and food webs. As a result, changes in the relative phenology, caused by changing cues of prey species, can drive diet switches of generalist consumers and potentially alter major energy pathways in ecosystems.

### (c) Changes in temporal variance and autocorrelation

Changing variance at different frequencies will alter the environment differently for organisms with different life histories. Environmental variables have become more temporally autocorrelated over the past 50 years, and these trends are expected to continue [172]. While increased temporal autocorrelation may increase environmental predictability, and therefore performance for some organisms [207], it may also reduce population persistence, because as the duration of poor conditions increases, refugia and rescue effects are diminished and extinction risk increases [32,208,209]. The effects of increased temporal variance in environmental variables will depend on the frequency at which variance increases and the life history of the organisms affected. For example, if variance increases at annual time scales, organisms with short generation times that are active only during the summer months may experience large changes to growth rates owing to multiple successive generations experiencing high summer temperatures. For longer-lived organisms whose reproductive cycle encompasses the whole year, if variance increases at the annual time scale then the increase in warm temperatures may be balanced (or not) by colder winter temperatures [172]. Alternatively, reduced variance at annual time scales, such as reduced differences between summer and winter temperatures in the form of milder winters, can substantially alter ecosystem structure and function. Changing community and ecosystem responses to milder winters are enhancing productivity and expanding growing seasons as climate changes in temperate and polar regions. This outcome is reducing the effects of extreme seasonal conditions and the life-history traits that allow organisms to reduce activity in winter.

## 9. Looking forward

Organisms in naturally variable environments exploit fluctuations and correlations among environmental variables to survive and persist. The ways in which they sense, communicate, anticipate and respond to environmental fluctuations determine patterns of biodiversity. Humans are changing patterns of auto- and cross-correlations in the environmental variables upon which cues are based. The extent to which these anthropogenic influences will alter the structure and function of ecosystems will depend on the mechanisms by which individuals respond to and anticipate fluctuations and adapt to changing fluctuation regimes. Here we have provided a framework that includes feedback and feedforward as different modalities of response and described how these mechanisms operate at multiple scales of biological organization. Recognizing that organisms employ a range of feedback and feedforward systems to mediate fitness suggests we must study the internal models they use to predict future ecological outcomes, and how they adapt to changing selective environments. One might expect the pace of evolutionary change to be generally faster in feedback systems compared to feedforward systems, and for some internal models to be more labile and adaptable than others, and this requires further study. An understanding of community responses to environmental change will require the study of the diversity of cues and internal models used by community members.

There is a high cost to ignoring the manner by which organisms and systems have adapted to fluctuating environments when assessing the effects of global change. Although it is a tall order to empirically measure yet another aspect of biotic responses to environmental change, we suspect that similarities and generalities in response types will be revealed, allowing them to be understood and predicted. If a feedforward mechanism exists, then an experiment that lacks appropriate cues may grossly mis-estimate the effects of environmental change (e.g. the loss of CO_2_ responses in fish when parental effects were allowed [174]). One way to probe the internal model of an organism would be to expose it to different types of cues in a controlled way, so as to identify the relevant cue. Manipulating the correlations between different environmental variables (e.g. temperature and oxygen, or light wavelength and depth) would reveal which signal and cues are important, and to what extent living systems can update their internal models when cues are no longer reliable. Manipulating – or considering how global change affects—the colour of environmental noise by adding variation (power) at different frequencies to elicit responses at the individual, population and community levels would allow us to understand how changes in fluctuations are amplified or dampened across trophic levels and how organisms with different life histories are influenced by fluctuations at different frequencies.

## 10. Conclusion

An outstanding challenge is to understand the degree to which feedback and feedforward mechanisms generate the diversity and dynamics of living systems. Explicitly considering the processes by which organisms respond to uncertainty about the future state of the environment may dramatically change our predictions of how living systems will respond and adapt to global environmental change. The task for ecologists is to discover the internal models that organisms use to anticipate environmental fluctuations, and how variation in these models among individuals and species governs their selection under environmental change, in the context of populations and communities.

## Data accessibility

Data and code to reproduce figures are available at https://doi.org/10.5281/zenodo.4058887.
